# Efficacy and Safety of Intravenous Vernakalant in Rapid Cardioversion of Recent Onset Atrial Fibrillation: A Retrospective Single-Centre Study

**DOI:** 10.7759/cureus.58616

**Published:** 2024-04-19

**Authors:** Abbas Hoteit, Mohamad B. Moumneh, Acile Nahlawi, Elsa Hebbo, Farah Abdulhai, Bernard Abi-Saleh, Maurice Khoury, Marwan Refaat

**Affiliations:** 1 Cardiology, American University of Beirut Medical Center, Beirut, LBN; 2 Heart and Vascular, Inova, Virginia, USA

**Keywords:** cardiac arrhythmias, atrial fibrillation, safety, efficacy, pharmacological cardioversion, vernakalant

## Abstract

Background: We use vernakalant, an intravenous anti-arrhythmic, to cardiovert paroxysmal atrial fibrillation (AF) into sinus rhythm. It is a relatively atrium-selective, early-activating potassium and frequency-dependent sodium channel blocker with a half-life of 2 to 3 hours. Due to concerns regarding its safety profile, it is not Food and Drug Administration (FDA)-approved.

Objective: This study aims to assess the efficacy of intravenous vernakalant in cardioversion of paroxysmal AF and the safety of its use.

Methods: Patients with paroxysmal AF who presented to the American University of Beirut Medical Center (AUBMC) between 2015 and 2020 and received vernakalant for cardioversion were included. Patients did not receive vernakalant if they had any of the following: QTc > 440 ms, heart rate < 50 bpm, acute coronary syndrome within the last 30 days, second- and third-degree atrioventricular (AV) block in the absence of a pacemaker, severe aortic stenosis (AS), use of intravenous antiarrhythmics (class I and class III) within four hours of vernakalant infusion, systolic blood pressure <100 mmHg, and heart failure (New York Heart Association (NYHA) III or NYHA IV class). The primary endpoint is conversion to sinus rhythm for at least one minute within 90 minutes of the start of the vernakalant infusion. The secondary endpoint included the presence of these side effects: bradycardia, QTc prolongation, AV block, ventricular arrhythmias, hypotension, taste alteration/dysgeusia, sneezing, nausea, vomiting, paresthesia, cardiogenic shock, or death.

Results: The study included 23 patients with paroxysmal AF (15 men, mean age 54 ± 14 years). Fourteen patients (61%) cardioverted to sinus rhythm within 90 minutes of the start of the Vernakalant infusion. Seven patients (30%) reverted to sinus rhythm within 15 minutes after the first infusion. After treatment with vernakalant, four patients (17%) developed sinus bradycardia, and four patients (17%) developed first-degree AV block. No patient had a QTc greater than 460 ms. None of the patients experienced sinus pauses, high-grade AV block, ventricular arrhythmias, hypotension, dysgeusia, sneezing, nausea, vomiting, paresthesia, cardiogenic shock, or death.

Conclusion: Vernakalant had 61% efficacy in the rapid cardioversion of paroxysmal AF to sinus rhythm, was well tolerated, and had a low rate of adverse events in our study population.

## Introduction

Atrial fibrillation (AF) is the most common cardiac arrhythmia seen in the emergency department and in clinical practice, with an estimated incidence of 73.4 and 49.9 cases per 100,000 person-years for male and female individuals, respectively [1]. Its prevalence is expected to increase significantly given the trend of aging populations and the subsequent increased burden of comorbidities such as coronary artery disease, hypertension, etc. [2]. Among adults aged 45 years or older, it has an estimated lifetime risk of one in three to five individuals [3]. These alarming numbers, coupled with the significant morbidity and mortality resulting from AF, make optimizing its management a necessity [4-6]. There are two main therapeutic approaches to treating AF, namely, rate control and rhythm control. There exists conflicting data regarding the superiority of a rate control strategy versus a rhythm control strategy. This approach is generally preferred among young patients who are highly symptomatic. It aims to improve quality of life by controlling symptoms, shortening hospital stays, and improving exercise tolerance [7-10]. Pharmacological cardioversion is one of the main treatment modalities to achieve rhythm control and restore sinus rhythm in recent-onset AF. Compared to electrical cardioversion, it offers several advantages, such as not needing general anesthesia or sedation and a lower risk of an immediate recurrence of AF. Its limitations revolve around the efficacy and safety of the different antiarrhythmic agents used. This makes it potentially the preferred treatment modality, provided an effective and safe antiarrhythmic agent is available [11]. Vernakalant recently emerged as a safe and effective rapid-acting antiarrhythmic drug for the conversion of recent-onset AF. A number of randomized clinical trials comparing its efficacy with a placebo and with other antiarrhythmic agents demonstrated its emergence. The European Union approved its use in 2010 [12]. Vernakalant is an atrial-selective antiarrhythmic agent that prolongs the effective refractory period of the atria. It acts as an inhibitor of atrial-selective potassium currents in addition to rate-dependent inhibition of atrial-predominant sodium channels. It is considered to have a low proarrhythmic risk due to its minimal effects on the ventricles [13]. Data on Vernakalant’s efficacy and safety remain limited. Adverse events associated with vernakalant include sensory disturbances, hypotension, bradycardia, arrthymias, and death in extreme cases. The aim of our study is to assess the efficacy of intravenous (IV) vernakalant in cardioversion of recent-onset AF (<48 hours) back to sinus rhythm and to determine its short- and long-term safety.

## Materials and methods

Definitions

Recent-onset AF was defined as a symptomatic episode that started within two days of presentation and receiving cardioversion therapy. Time to cardioversion was considered from the moment sinus rhythm was achieved until patient discharge. Persistent AF beyond two hours of vernakalant dose administration was considered a failure of pharmacological therapy.

Patients

We conducted a single-center retrospective study in the Emergency Department and the Cardiac Care Unit at the American University of Beirut Medical Center (AUBMC). Patients aged above 18 years who presented with a diagnosis of recent-onset AF and received IV vernakalant for pharmacological cardioversion between January 1, 2015, and December 1, 2020, were identified. A total of 23 patients who received IV vernakalant as their sole source of pharmacological cardioversion were recruited into our study.

Data collection

Relevant patient data were collected from patient records. The hospital uses an electronic medical records system, which includes the patients’ medications, ECGs, vital signs, and laboratory tests. Records were reviewed using an IRB-approved data collection sheet to collect data on the patient’s clinical course, previous medical history, medications, and treatment plans for AF.

Vernakalant cardioversion

Vernakalant was administered intravenously with an initial dose of 3.0 mg/kg (up to a maximum of 339 mg) during a 10-minute infusion. If sinus rhythm was not achieved within 15 minutes, a second dose of 2.0 mg/kg (up to a maximum of 226 mg) was administered during a 10-minute infusion. Patients were monitored during the infusion and for at least two hours after conversion to sinus rhythm to document treatment success. Patients who received concurrent pharmacological cardioversion with an IV formulation of other anti-arrhythmic drugs were not included in our study. Patients who failed pharmacological cardioversion with vernakalant underwent subsequent electrical cardioversion, according to the judgment of the treating/admitting physician.

Study objectives and endpoints

The primary endpoint of the study was the efficacy of Vernakalant in cardioversion to sinus rhythm within 90 minutes of drug administration and discharge in sinus rhythm. Secondary endpoints were side effects and adverse events after drug administration. These included nausea, paresthesia, taste alteration, sneezing, bradycardia <50 bpm, AV block, QT prolongation, ventricular arrhythmias, cardiac shock, and in-hospital death.

Statistical analysis

The statistics were analyzed using IBM Statistics for Windows (IBM Corp., Armonk, NY). The data are presented as mean values ± standard deviation (SD) or frequency percentages.

## Results

Baseline characteristics

Table 1 presents the demographic data, baseline characteristics, and medical history of the subjects. Patients had a mean (±SD) of 54 (±14.4) years. Of the study population, 52% had already experienced an episode of AF prior to admission, during which they were administered vernakalant. Prior to the administration of vernakalant, the duration of the AF episode was 309 (±333) minutes. A prior history of electrical cardioversion of AF was performed in 22%, and 9% of individuals had an ablation performed for AF.

**Table 1:** Demographic data of our study population AF: atrial fibrillation; CAD: coronary artery disease; HTN: hypertension; MI: myocardial infarction.

Characteristics	Patient number (%)
Age (years)	54 ± 14.4 years
Men	15 (65)
Episode duration	309 ± 333 minutes
Diabetes	3 (13)
HTN	7 (30)
CAD	3 (13)
Prior MI	1 (4)
Left ventricular hypertrophy	3 (13)
First episode of AF	11 (48)
Paroxysmal AF	11 (48)
Permanent AF	1 (4)
History of ablation	2 (9)
History of cardioversion	5 (22)
History of atrial flutter	2 (9)
History of other arrhythmias	2 (9)

Efficacy

As shown in Table 2, within 15 minutes of the administration of the first dose of vernakalant, 30.5% of subjects reverted to sinus rhythm. At 90 minutes, around 61% of patients reverted to sinus rhythm for at least one minute. The average time needed to revert to sinus rhythm was 28 (±) 19 minutes after administration of the first dose of vernakalant.

**Table 2:** Primary and secondary endpoints

Endpoints	Patient number (%)
Sinus rhythm restored within 15 minutes	7 (30.5%)
Sinus rhythm restored within 90 minutes after start of infusion (for at least 1 minutes)	14 (31%)
Time to sinus rhythm	28 ± 19 minutes
QT prolongation	2 (9%)
Sinus bradycardia	4 (17%)
AV block	4 (17%)
Nausea	0 (0%)
Paresthesia	0 (0%)
Hypotension	0 (0%)
Cardiac shock	0 (0%)
Ventricular arrhythmias	0 (0%)
Torsades de pointes	0 (0%)

Safety profile

After treatment with vernakalant, four patients (17%) developed sinus bradycardia, and four patients (17%) developed first-degree AV block. Of the subjects, 9% developed an increase in QTc interval above baseline and above 440 ms, but none had QTc greater than 460 ms. Sinus bradycardia and first-degree AV block reverted after 24 hours. None of the patients experienced sinus pauses, high-grade AV block, ventricular arrhythmias, hypotension, dysgeusia, sneezing, nausea, vomiting, paresthesia, cardiogenic shock, or death.

## Discussion

Pharmacological cardioversion in patients with the recent onset of AF is crucial to the rapid restoration of sinus rhythm [14]. Identifying a safe and efficient intravenous antiarrhythmic drug that can achieve such cardioversion is coveted. In our retrospective study, 23 patients were treated with 3 mg/kg IV vernakalant over 10 minutes and a second dose of 2 mg/kg when necessary. Seven patients (30.4%) converted from AF to sinus rhythm within 15 minutes, while 14 (61%) converted from AF to sinus rhythm for at least one minute within 90 minutes after the start of the infusion. Our conversion rate was similar to rates recorded by other trials. A phase III study compared the efficacy of vernakalant to that of amiodarone in subjects with recent onset AF and reported a superior role of vernakalant in conversion from AF to sinus rhythm (vernakalant’s 51.7% to amiodarone’s 5.7%) [15]. Similarly, a randomized control trial by Simon et al. demonstrated vernakalant’s superior role in sinus rhythm restoration compared to ibutilide (69% versus 43%), while the study by Pohjantahti-Maaroos et al. demonstrated its superior role in restoring rhythm compared to flecainide (67% vs. 46%) [16,17].

As for studies comparing conversion rates to sinus rhythm in patients receiving vernakalant versus those receiving a placebo, results showed rates of 37.6% and 2.6%, respectively, in the phase III randomized placebo-controlled trial by Roy et al., 47% and 14%, respectively, in the randomized, double-blind, placebo-controlled trial of noncritically ill patients having AF after cardiac surgery by Kowey et al., 45.7% and 1.5%, respectively, in the phase 3b randomized controlled trial by Beatch and Mangal, 53% and 5%, respectively, in the randomized controlled trial by Roy et al., and 39.8% and 3.3%, respectively, in the study by Pratt et al. [18-22].

Concerning safety and severe adverse effects (SAE), 4 of our 23 patients (17.4%) experienced bradycardia, 4 (17.4%) experienced AV block, and 2 (8.7%) experienced QT prolongation from baseline. The study by Kowey et al. identified bradycardia as an SAE, with 10 out of 107 (9.1%) vernakalant patients experiencing bradycardia within two hours of dosing. There was a reported 5% increase in QTc from baseline as well, while 14 (13.1%) of their vernakalant patients experienced hypotension, and 4 (3.7%) experienced ventricular tachycardia [19]. In our study, both SAEs of hypotension and ventricular tachycardia were not recorded, likely due to the small sample size of our study. Bradycardia and prolonged QTc were also reported in the superiority study comparing vernakalant and amiodarone, with 5 out of the 166 (2.6%) vernakalant patients experiencing symptomatic bradycardia <40/minutes, while QTc increased by 25 ms from baseline [15]. Bradycardia was also seen in the SPECTRUM study (0.5%), the superiority study comparing vernakalant and ibutilide, where 2 out of 49 (4.1%) had bradycardia, and the study by Rudiger et al. on critically ill patients, where 2 of their 32 patients (6.25%) experienced bradycardia [16,23,24]. In the latter study, 11 patients (34%) experienced hypotension, and 0.1% experienced hypotension in the SPECTRUM study, with this SAE not recorded in our study as previously mentioned [23,24]. An increase in QTc was also detected in the study by Pratt et al. (20-25 ms increase from baseline) and in the phase III randomized placebo-controlled trial by Roy et al. (6% increase from baseline) [18,22]. No ventricular tachycardia or torsades de pointes were seen in our vernakalant patients, which corresponds with the SPECTRUM, Rudiger, and Kowey studies. 

Limitations

The main limitations of the study are the retrospective design and the limited number of patients recruited from a single tertiary center. The results should be taken with caution. Additional and larger studies are necessary to confirm the efficacy and safety of vernakalant use in cardioversion in recent-onset AF.

## Conclusions

IV vernakalant showed a relatively well-tolerated safety profile and moderate efficacy in cardioversion. Our cohort showed a low rate of SAE. We resolved the adverse events within 24 hours. Vernakalant might be a safe and effective alternative means of pharmacological cardioversion in patients with recent-onset AF. However, due to the limitations of our study, it is too early to draw conclusions and give recommendations. Further studies are needed to look into this topic.
